# Ovarian Dropsy, Treated by Tapping and Injection of Solution of Iodine

**Published:** 1847-08

**Authors:** 


### MEDICAL EXAMINER,

AND

### RECORD OF MEDICAL SCIENCE.

NEW SERIES.—No. XXXII.—AUGUST, 1847.

---

### ORIGINAL COMMUNICATIONS.

### *Ovarian Dropsy, treated by tapping and injection of Solution of Iodine.*

In the number of the Medical Examiner for June, 1846, we published an account of a case of Ovarian Dropsy by Dr. B. A. Allison, of Spencer, Indiana, in which, after repeated tapping, he injected a solution of iodine into the sac. The novelty and boldness of the operation and its apparent success, excited much attention at the time, and the paper was extensively copied, with various comments. Under these circumstances, the following extract of a letter received from Dr. Allison, dated June 11, 1847, will be read with interest, and perhaps lead to a further trial of the practice which in his hands seems to have been eminently successful.

"Soon after the date of my letter to you containing an account of the case of Mrs. C—— (May, 1846,) her menses returned, and have continued regular ever since, and she has enjoyed as good health as any woman in the neighbourhood, excepting that the orifice, where tapping was performed, is not entirely closed yet, and occasionally discharges a few drops of healthy pus. A curious feature occurred in her case soon after she began to take exercise—her lower extremities became affected with numerous large, spreading and obstinate ulcers, which yielded, however, in time, to the bandage, and occasional application of the citrine ointment."

